# Non-surgical nursing care for tumor patients: an overview of sedation, analgesia, and recent innovations

**DOI:** 10.3389/fonc.2024.1322196

**Published:** 2024-09-17

**Authors:** Wei Wei, Pan Wang, Pan Qing, Zhang Li, Qi He

**Affiliations:** ^1^ Department of Anesthesiology, Affiliated Sport Hospital of CDSU, Chengdu, Sichuan, China; ^2^ Department of Pain, Zibo Central Hospital, Zibo, Shandong, China; ^3^ Department of Geriatric Orthopaedics II, Sichuan Orthopaedic Hospital, Chengdu, Sichuan, China

**Keywords:** tumor sedation, tumor analgesia, non-surgical nursing care, pain management, nonpharmacological interventions, palliative care, recent innovations, symptom management

## Abstract

With the increasing prevalence of tumors, effective symptom management has emerged as a cornerstone of patient care. While surgical interventions remain pivotal, non-surgical nursing methods have gained prominence in providing relief from pain, discomfort, and other tumor-related symptoms. This review delves into the various non-surgical approaches employed, emphasizing tumor sedation and analgesia. We discuss the array of non-pharmacological and pharmacological strategies, shedding light on their indications, contraindications, and potential side effects. Furthermore, the importance of addressing individual differences in pain perception and the ethical considerations in symptom management are highlighted. We conclude by providing insights into the recent innovations in the field, emphasizing the need for personalized and comprehensive care to enhance patients’ quality of life. Tumor sedation, Tumor analgesia, Non-surgical nursing care, Pain management, Non-pharmacological interventions, Palliative care, Recent innovations, Symptom management.

## Highlights

- Non-Surgical Nursing Methods: Pivotal shift from surgical to non-surgical approaches in tumor symptom management, expanding care options for patients.
- Comprehensive Strategies: Balanced examination of non-pharmacological and pharmacological methods, offering a holistic view on pain and symptom management.
- Individual Pain Perception: Emphasis on the variability in pain perception, advocating for personalized treatment plans.
- Ethical Considerations: Integration of ethical perspectives in symptom management, addressing complex healthcare dilemmas.
- Innovations in the Field: Insights into recent advancements and future possibilities in tumor symptom management, guiding research and clinical practice.

## 1 Introduction

With the increasing prevalence of tumors, effective symptom management has emerged as a cornerstone of patient care. While surgical interventions remain pivotal, non-surgical nursing methods have gained prominence in providing relief from pain, discomfort, and other tumor-related symptoms. This review delves into the various non-surgical approaches employed, emphasizing tumor sedation and analgesia. We discuss the array of non-pharmacological and pharmacological strategies, shedding light on their indications, contraindications, and potential side effects. Furthermore, the importance of addressing individual differences in pain perception and the ethical considerations in symptom management are highlighted. We conclude by providing insights into the recent innovations in the field, emphasizing the need for personalized and comprehensive care to enhance patients’ quality of life (1).

In recent years, the medical community has witnessed a renewed emphasis on the significance of pain and discomfort management in patients with tumors (2). As the global incidence of tumors continues to rise, so does the urgency to address the multifaceted challenges these patients face (3). Pain, often a relentless companion of tumor growth, has profound implications not only on a patient’s physical well-being but also on their psychological health, impacting facets of daily life, from sleep quality to interpersonal relationships (4). Recent research underscores that unmanaged or poorly managed pain can lead to heightened levels of anxiety, depression, and even decreased survival rates in some cases (5). In this evolving landscape, non-surgical nursing care has emerged as a critical player (6). These approaches, ranging from pharmacological interventions to holistic care models, have showcased potential in not only alleviating pain but also in enhancing the overall quality of life for patients (7). Recent studies indicate that patients receiving comprehensive non-surgical care often report improved outcomes, reduced hospital stays, and a more positive prognosis (8). As the dynamics of tumor care shift towards a more patient-centric model, the pivotal role of non-surgical nursing care in bridging the gap between medical intervention and enhanced patient well-being cannot be overstated.

## 2 Tumor sedation

A study on high-quality care for postoperative inflammation and prognosis in advanced non-small cell lung cancer (NSCLC) patients suggests that high-quality care significantly reduces hospitalization duration, improves postoperative inflammation management, symptom control, and quality of life when compared to patients receiving standard care. In comparison to regular care, high-quality care reduces anxiety, depression levels, and psychological distress in postoperative advanced NSCLC patients. The results indicate that high-quality care prolongs the survival time and reduces the recurrence rate of postoperative advanced NSCLC patients (9). Tumor sedation has garnered considerable attention in contemporary oncological care, reflecting its significance in improving patient comfort and quality of life (10). Defined as the deliberate use of medications to relieve extreme symptoms, especially refractory pain and distress in advanced cancer patients (11), tumor sedation aims to achieve a state where the patient’s consciousness is reduced while ensuring their comfort and dignity (12). The core objective of this approach is to mitigate suffering, especially when other treatments fail to provide relief. In recent times, advancements in pharmacology have introduced new agents and refined protocols that offer more controlled and individualized sedation, minimizing potential side effects (13). Moreover, the development of precision monitoring tools aids healthcare professionals in ensuring that sedation is maintained at optimal levels (14), allowing the patients to interact with their loved ones and respond to their environment, even if minimally. These recent strides in tumor sedation techniques underline the medical community’s commitment to enhancing the end-of-life experience for patients, emphasizing comfort, autonomy, and humanity. **Table 1** provides a comparison of medications used for tumor sedation, including Midazolam, Propofol, and Dexmedetomidine, along with their respective mechanisms of action, common uses, and potential side effects.

**Table 1:** Comparison of medications used for tumor sedation.

Medication Name	Mechanism of Action	Common Uses	Potential Side Effects
Midazolam	Benzodiazepine	Short-term sedation	Drowsiness, respiratory depression
Propofol	GABA receptor agonist	Induction and maintenance of anesthesia	Hypotension, bradycardia
Dexmedetomidine	Alpha-2 adrenergic receptor agonist	Sedation in ICU settings	Bradycardia, dry mouth

The landscape of tumor sedation has evolved considerably in the past few years, enriched by a confluence of innovative methods and advanced medications tailored to meet the unique needs of tumor patients (15). Traditionally, benzodiazepines, such as midazolam, have been a mainstay for sedation due to their rapid onset and short duration of action (16). However, the introduction of newer agents, including propofol and dexmedetomidine, has expanded the therapeutic arsenal (17). Propofol, in particular, offers rapid sedation with a smooth recovery profile, while dexmedetomidine, an alpha-2 adrenergic receptor agonist, provides sedation without causing respiratory depression (18). Beyond pharmacological advances, non-pharmacological techniques, like progressive muscle relaxation and guided imagery, have gained traction as adjunctive therapies, enhancing the sedative experience while minimizing drug-related side effects (19). Furthermore, the integration of continuous monitoring systems allows for real-time adjustments of sedative doses, ensuring optimal sedation levels and patient safety (20). These advancements underscore a holistic and patient-centered approach, where the choice of method and medication is intricately aligned with the patient’s clinical status, preferences, and the intended depth of sedation.

In the ever-evolving realm of oncology, clear guidelines for when and when not to employ tumor sedation are pivotal to ensuring patient safety and optimal outcomes (21). Recent consensus and evidence-based guidelines have delineated the indications for tumor sedation more explicitly (22). Primarily, it is reserved for patients with refractory symptoms—those distressing symptoms unresponsive to standard medical interventions, such as uncontrolled pain, agitated delirium, or severe dyspnea (11). The goal is to alleviate suffering when other treatments fall short. In certain end-of-life scenarios, sedation may also be employed to ensure a peaceful transition for terminal patients (23). On the flip side, contraindications have been more rigorously defined (24). Tumor sedation is generally avoided in patients with reversible causes of distress, those who might benefit from other interventions, or where the intent might be misunderstood or misconstrued by the patient’s family (25).

A systematic review and meta-analysis studying the impact of enhanced care in liver cancer patients indicate that enhanced care significantly improves patient anxiety, depression, and quality of life. Most liver cancer patients receiving enhanced care are highly satisfied with their quality of life. Furthermore, the analysis also demonstrates significant improvements in patients’ physical functioning and overall activity scores due to enhanced care (26).

A study on the effectiveness of patient-reported personalized symptom management in liver cancer intervention therapy found that patients who received personalized management experienced significantly milder symptoms such as pain, nausea, anxiety, and fatigue compared to those receiving standard care. Moreover, patients in the intervention group showed a significant improvement in Karnofsky performance scores and satisfaction with care (27) **Table 2** outlines various alternative therapies and their potential benefits, including Acupuncture, Aromatherapy, and Music Therapy. Additionally, certain medications used for sedation may be contraindicated in patients with specific allergies or organ dysfunctions. With advancements in diagnostic tools and a better understanding of patient physiology, the decision-making process around tumor sedation has become more refined, ensuring that it is applied judiciously and benefits those truly in need. **Table 3** offers strategies to manage common side effects of pain medications, such as constipation, respiratory depression, and gastrointestinal distress. These strategies are particularly useful when dealing with opioids, high doses of opioids, and NSAIDs, respectively.

**Table 2:** Alternative therapies and their potential benefits.

Therapy Type	Description	Potential Benefits
Acupuncture	Traditional Chinese therapy using needles	Pain relief, reduced nausea
Aromatherapy	Use of essential oils for therapeutic purposes	Stress reduction, improved sleep
Music Therapy	Use of music interventions to address physical, emotional, cognitive, and social needs	Mood enhancement, reduced anxiety

**Table 3:** Strategies to manage common side effects of pain medications.

Side Effect	Cause	Management Strategy	Additional Notes
Constipation	Opioids	Laxatives, increased fiber intake, hydration	Monitor for abdominal pain or bloating
Respiratory Depression	High doses of opioids	Patient monitoring, naloxone	Educate patients about the risks
Gastrointestinal Distress	NSAIDs	Co-prescription with proton pump inhibitors	Encourage patients to take with food

Lastly, a cluster randomized clinical trial explored the effectiveness of primary palliative care interventions provided by oncology nurses, which did not improve patients’ quality of life, symptom burden, or mood symptoms within 3 months. However, the study highlighted that higher-dose interventions may be beneficial for most advanced cancer patients who lack access to palliative care specialists (28).

## 3 Tumor analgesia (pain management)

In the contemporary landscape of oncology, the emphasis on pain management in tumor patients has never been more pronounced (29). Pain, often chronic and debilitating, is an all-too-common companion for many tumor patients, profoundly impacting their physical and psychological well-being (30). Recent studies underscore that inadequately managed pain not only diminishes the quality of life but also can exacerbate tumor progression, potentially influencing metastasis and immune suppression (31). The physiological stress induced by persistent pain can lead to elevated cortisol levels, which, in turn, can have detrimental effects on the body’s ability to fight off tumor cells (32). Moreover, effective pain management is intricately linked to improved patient outcomes, including better treatment adherence, reduced hospitalization durations, and enhanced overall survival rates (33). The realm of tumor analgesia has also witnessed a paradigm shift towards a holistic model, where pain is viewed not merely as a physiological symptom but as a complex interplay of emotional, social, and psychological factors (34). This comprehensive approach underscores the critical need for personalized pain management strategies, ensuring that patients can lead a life with dignity, comfort, and hope.

The pharmacological landscape of tumor analgesia has witnessed remarkable advancements in recent years, offering patients a broader and more tailored spectrum of pain relief options (35). Opioids, such as morphine, oxycodone, and fentanyl, remain at the forefront of managing moderate to severe cancer pain (36). Their effectiveness is, however, often counterbalanced by concerns of tolerance, addiction, and side effects like constipation and respiratory depression (37). To address these challenges, extended-release formulations and targeted delivery systems have been developed, optimizing pain control while minimizing adverse effects (38). Parallelly, non-opioids like acetaminophen and NSAIDs provide relief for milder pain and can synergistically enhance opioid efficacy (39). Adjuvant analgesics, including anticonvulsants and antidepressants, have emerged as pivotal players, especially for neuropathic pain, a type of pain frequently associated with tumors and cancer treatments (40). The recent emphasis on personalized medicine has also fostered innovations in drug delivery (33). While oral administration remains common, the rise of transdermal patches, intravenous infusions, and even implantable drug delivery systems cater to specific patient needs (41), ensuring consistent pain relief while reducing systemic side effects (42). Collectively, these advancements underscore a commitment to a multifaceted and patient-centric approach to tumor analgesia, aiming for optimal pain relief with the least possible inconvenience or discomfort to the patient.

The management of pain in tumor patients, while imperative, is often accompanied by a range of medication-induced side effects that can pose substantial challenges to both patients and healthcare providers (43). Recognizing this, recent advances have been directed towards not just enhancing analgesic efficacy but also mitigating these adverse effects (44). For opioids, constipation remains a predominant concern; the introduction of peripherally-acting mu-opioid receptor antagonists, such as naloxegol, has been transformative in managing opioid-induced constipation without affecting central pain relief (45). Respiratory depression, another critical opioid-related side effect, is now better managed with the advent of naloxone nasal sprays and injectables, offering rapid reversal in emergent situations (46). To combat the gastrointestinal side effects of NSAIDs, co-prescription with proton pump inhibitors or the use of selective COX-2 inhibitors has gained traction (47). Furthermore, the practice of rotating opioids, a technique where one opioid is substituted for another, has shown promise in reducing tolerance and side effects (48). Patient education, regular monitoring, and the use of digital health platforms for real-time symptom tracking and reporting have also become integral to a proactive management approach (49). These strategies, born out of a blend of technological innovation and refined pharmacological understanding, represent a concerted effort to ensure that pain management is both effective and tolerable for tumor patients.

## 4 Other non-surgical nursing care methods

In the realm of tumor care, the surge of interest in alternative therapies has redefined the boundaries of non-surgical nursing interventions (50). While conventional treatments remain foundational, a growing body of evidence suggests that alternative therapies can play a significant role in enhancing patient well-being and potentially alleviating tumor-related symptoms. Acupuncture, a traditional Chinese medical practice, has made notable inroads in the oncological setting (51). Recent studies indicate its efficacy in managing chemotherapy-induced nausea, postoperative pain, and even cancer-related fatigue (52). Aromatherapy, the therapeutic use of essential oils, has been spotlighted for its potential in reducing anxiety, improving sleep, and enhancing overall mood in tumor patients (53). Lavender, chamomile, and frankincense are among the oils frequently utilized (54). Meanwhile, music therapy, a confluence of art and science, has emerged as a potent tool (55). Tailored musical interventions, whether passive listening or active participation, have been linked to reduced pain perception, decreased levels of stress hormones, and improved emotional well-being (56). While these therapies might not replace conventional treatments, their integration into the holistic care model underscores a broader understanding of patient needs, emphasizing not just physical health but also psychological and emotional harmony.

As the complexities of tumor care continue to unfold, addressing multifaceted tumor-related symptoms has become paramount. Beyond pain, symptoms like fatigue, nausea, and cognitive disturbances can severely impede a patient’s quality of life (57). In recent years, targeted interventions have been developed to combat these challenges (58). Fatigue, often cited as one of the most debilitating symptoms by tumor patients, is now being addressed through a combination of pharmacological agents, like modafinil, and non-pharmacological strategies (59), such as graded exercise therapy and cognitive behavioral therapy. Nausea, particularly post-chemotherapy, has seen significant advancements in management (60). The introduction of newer antiemetic drugs, like the NK1 receptor antagonists and olanzapine, has improved control rates, especially in patients undergoing highly emetogenic treatments (61). Additionally, techniques like progressive muscle relaxation and guided imagery have shown promise in alleviating nausea (62). For cognitive disturbances or “chemo brain,” interventions ranging from cognitive rehabilitation programs to mindfulness meditation have been explored (57). The integration of digital health tools, offering real-time symptom tracking and personalized interventions, is also revolutionizing the approach to symptom management (63). These innovations underscore the evolving nature of tumor care, where a nuanced understanding of symptoms and a multi-pronged approach to their management ensure that patients lead a life not just free of pain, but also enriched in well-being (64).

Palliative care, once perceived as a last-resort intervention for terminal patients, has undergone a paradigm shift in recent years (65). Today, it’s recognized as an integral component of comprehensive tumor care, introduced early in the disease trajectory and woven seamlessly alongside curative treatments (66). The primary objective of modern palliative care is to enhance the quality of life, addressing physical symptoms, emotional distress, spiritual concerns, and social challenges (67). Recent studies have illuminated its profound impact: patients receiving early palliative care interventions report better symptom control, improved mood, and even, in some cases, extended survival (68). This holistic approach emphasizes person-centered care, focusing on the patient’s goals, values, and preferences (69). Technological advancements, such as telemedicine platforms, have further broadened the reach of palliative care, ensuring that even those in remote areas or with limited mobility can access these crucial services (70). Interdisciplinary collaboration, encompassing doctors, nurses, therapists, and counselors, ensures that every facet of a patient’s well-being is addressed (71). As the medical community continues to understand the complexities of tumor care, the role of palliative care as a beacon of comfort, dignity, and hope in the patient’s journey becomes ever more central.

## 5 Challenges in non-surgical nursing care

Navigating the intricacies of non-surgical nursing care for tumor patients has brought to light the profound individual variability in pain perception and tolerance (72). Recent research underscores that pain, far from being a uniform experience, is deeply personal, shaped by a mosaic of genetic, physiological, psychological, and cultural factors (73). Genetic polymorphisms can influence the metabolism of analgesic drugs, leading some patients to require higher or lower doses for effective relief (74). Additionally, psychological states, such as anxiety or depression, can modulate pain perception, often amplifying the experience of pain (75). Cultural beliefs and past experiences can also play pivotal roles in shaping how pain is expressed and tolerated (76). Addressing these individual nuances has posed significant challenges in standardizing care (77). However, the emergence of precision medicine and pharmacogenomics offers promise (33). Tailored pain management strategies, based on an individual’s genetic makeup and holistic assessment, are being explored (78). Furthermore, interdisciplinary approaches, combining medical, psychological, and socio-cultural insights, are being employed to ensure a more comprehensive understanding and management of pain (79). While the path is fraught with challenges, the commitment to individualized, patient-centric care remains unwavering in the face of these complexities (80).

The multifaceted pharmacological landscape of tumor care, while pivotal in managing symptoms, has brought to the fore the intricate challenge of potential drug interactions and side effects (81). As patients often receive a plethora of medications, ranging from chemotherapeutic agents to adjuvant analgesics, the risk of unforeseen interactions escalates (82). These interactions can potentiate drug toxicity, diminish therapeutic efficacy, or introduce new, unanticipated side effects (83). To address this, recent advancements have prioritized comprehensive drug interaction databases and real-time monitoring systems (84). Advanced algorithms, informed by vast pharmacological data, now provide clinicians with immediate alerts if a proposed medication regimen risks harmful interactions (85). Alongside this, there’s a growing emphasis on pharmacovigilance, where systematic post-market monitoring of drugs ensures timely detection and mitigation of side effects (86). Patient education has also emerged as a cornerstone; empowering patients with knowledge about potential side effects and fostering open communication channels allows for early detection and intervention (7). Additionally, the rise of personalized medicine, where treatment regimens are tailored based on genetic and metabolic profiles, offers hope in minimizing adverse reactions (87). Through a blend of technology, vigilant monitoring, and patient engagement, the medical community is steering towards safer and more effective pharmacological management in tumor care (88).

Amidst the complex dynamics of pain and symptom management in tumor care, ethical considerations have risen to the forefront (89), demanding a delicate balance between alleviating suffering and ensuring patient autonomy and dignity (90). The recent discourse has intensified around issues like over-prescription of opioids, where the intent to relieve pain may inadvertently lead to dependence or misuse (91). Consequently, there’s a pressing call for clear guidelines, informed consent, and regular monitoring to ensure opioids are used judiciously (92). Similarly, the decision to initiate, withhold, or withdraw treatments, especially in end-of-life scenarios, is fraught with ethical dilemmas (93). Shared decision-making models, emphasizing transparent communication between patients, families, and healthcare providers, are gaining prominence (94). These models prioritize the patient’s values, beliefs, and preferences, ensuring that interventions align with their overall well-being and life goals (95). Furthermore, the potential disparities in access to pain and symptom management resources, especially in underserved populations, have raised ethical concerns about equitable care (96). Efforts are underway to bridge these gaps through policy reforms and community outreach (97). As the medical community navigates these ethical waters, the commitment remains clear: to offer compassionate, respectful, and individualized care, always placing the patient’s holistic well-being at the heart of every decision.

## 6 Recent advances and innovations

The last few years have ushered in a renaissance of innovation in non-surgical tumor care, driven by groundbreaking research and technological advancements (98).

In support of the viewpoint advocating early specialized palliative care, the results of this study provide compelling evidence. While the CONNECT program did not significantly improve patients’ quality of life and symptom burden within 3 months, this finding underscores the challenges faced by current oncology patients. Many patients lack access to early specialized palliative care, which may impact their quality of life and symptom management. However, it’s worth noting that the CONNECT program showed a greater effect in patients who completed the full course, suggesting that high-dose primary palliative care may be particularly beneficial for certain patients. Therefore, we recommend that despite the current results not fully endorsing the effectiveness of early specialized palliative care, future research should focus more on high-dose primary palliative care interventions to meet specific patient needs and enhance their quality of life (99).

At the nexus of this evolution lies the burgeoning field of digital health, with wearable sensors and telemedicine platforms offering real-time symptom monitoring and personalized interventions (100). These tools, harnessing the power of artificial intelligence and big data analytics, enable clinicians to preemptively address symptoms, enhancing patient comfort and reducing hospitalizations (101). Another significant leap has been in the realm of pharmacogenomics, where individualized drug regimens, tailored to a patient’s genetic makeup, promise optimized therapeutic effects with minimized side effects (102).

The research on depression and anxiety among people living with and beyond cancer emphasizes the psychological well-being of cancer patients, particularly highlighting significant variations in anxiety and depression levels across different stages of cancer treatment (103). These differences profoundly impact the quality of life for patients. Throughout the process of cancer treatment, considering psychological health factors becomes crucial, especially in pain management and enhancing patient comfort. The paper on “Stress and Cancer” delves into the roles of psychological therapy and medication in managing cancer-related psychological stress, depression, and anxiety (104–106). These studies also mention emerging psychological treatment methods, such as tailored psychological interventions for advanced cancer patients. This research supports your perspective of emphasizing personalized treatment and comprehensive care in managing cancer symptoms. Therefore, it is imperative to focus not only on patients’ physical health but also on their psychological well-being.

Moreover, Wang et al.’s meta-analysis provides evidence of the relationship between depression, anxiety, and cancer incidence and mortality rates among various cancer types (107). The study results indicate a significant correlation between depression and anxiety with cancer incidence, cancer-specific mortality rates, and overall mortality rates among cancer patients. These findings once again underscore the pivotal role of psychological states in cancer treatment and prognosis, especially in pain management and improving the quality of life for patients. Other discussion explores how psychological stress affects tumor development through biobehavioral pathways (108). This review article emphasizes the close connection between psychological well-being and the management of tumor-related symptoms and patient care. While clinical evidence regarding the relationship between psychological stress and cancer may be inconsistent, animal studies suggest that prolonged psychological stress can significantly promote tumor progression. This discovery underscores the need to prioritize psychological health management alongside physical treatment in cancer care to provide holistic care and enhance the overall quality of life for patients.

Additionally, the exploration of neuromodulation techniques, such as transcranial magnetic stimulation, offers novel avenues for managing refractory pain and other tumor-related symptoms (109). Non-invasive brain stimulation methods are being researched for their potential in modulating pain pathways, offering relief without drugs (110). On the holistic front, integrative therapies combining Western medicine with traditional practices, such as yoga and mindfulness meditation, are gaining empirical validation (111), showcasing their efficacy in enhancing overall well-being. As the tapestry of non-surgical tumor care continues to expand and diversify, it’s clear that the future holds a multidimensional approach, seamlessly blending technology, pharmacology, and holistic care to revolutionize the patient experience (112). The horizon of non-surgical tumor care is brimming with possibilities, shaped by an interplay of technological innovation, scientific discovery, and evolving patient needs (113). One of the most anticipated advancements is the fusion of precision medicine with artificial intelligence, enabling predictive modeling of individual patient responses to various treatments, thereby optimizing therapeutic outcomes (114). This synergy promises to tailor interventions not just based on genetic profiles, but also by analyzing real-time physiological, behavioral, and environmental data (115). Another promising avenue is the exploration of bioelectronic medicine, harnessing the potential of electrical signals within the body to modulate pain and other symptoms without the use of drugs (116). As our understanding of the human microbiome deepens, there’s growing optimism about leveraging its potential to modulate pain and inflammation, offering novel therapeutic interventions (117). On the holistic front, there’s a palpable momentum towards integrating traditional healing practices from various cultures into mainstream care, backed by rigorous scientific validation. Additionally, the role of virtual and augmented reality in pain management and patient education is an emerging area of interest. As these innovations coalesce, the future of non-surgical tumor care envisions a holistic, integrated, and patient-centric model that transcends boundaries, ensuring the best possible quality of life for patients.

## 7 Conclusion

In reflection, the evolving landscape of tumor care underscores the undeniable significance of non-surgical nursing interventions in managing the multifaceted challenges faced by patients. Beyond the immediate relief from pain and discomfort, these interventions play a pivotal role in enhancing the overall quality of life, influencing physical well-being, psychological health, and social interactions. Recent advancements, whether technological, pharmacological, or holistic, have all converged towards one central theme: the importance of personalization. Recognizing that each patient’s journey with a tumor is unique, the emphasis has shifted towards tailored interventions that account for individual genetic, physiological, and emotional nuances. Furthermore, the move towards a more integrated and comprehensive care model, which seamlessly blends traditional and innovative practices, is a testament to the medical community’s commitment to ensuring that every patient receives the best possible care. As we navigate the complexities of tumor care, the value of a patient-centric, compassionate, and holistic approach remains at the heart of all endeavors, guiding the future trajectory of non-surgical interventions.
